# Diagnostic Challenge: A Report of Two Adult-Onset Still's Disease Cases

**DOI:** 10.1155/2017/3768603

**Published:** 2017-09-25

**Authors:** Sakunee Niranvichaiya, Daranporn Triwongwaranat

**Affiliations:** Department of Dermatology, Faculty of Medicine Siriraj Hospital, Mahidol University, Bangkok, Thailand

## Abstract

This study reports two adult-onset Still's disease (AOSD) cases that met both Yamaguchi's and Fautrel's criteria and that presented with notable clinical manifestations. One case presented with atypical dermographism-like rash with an extremely high ferritin level. The other case presented with typical salmon-pink maculopapular rash but had atypical positive rheumatoid factor. This suggests that although negative rheumatoid factor is one of the criteria used for the diagnosis of AOSD, a positive rheumatoid factor result does not exclude AOSD. Beside a classic rash, characterized by transient salmon-pink maculopapular rash, we also find atypical dermographism-like rash. These findings remind us that there exist various types of rash from AOSD.

## 1. Introduction

Adult-onset Still's disease (AOSD) is a rare systemic inflammatory condition with unknown etiology that is characterized by high spiking fever, arthritis, typically salmon-pink maculopapular rash, leukocytosis with neutrophilia, and multiple organs' involvement [1]. The diagnosis can be made by excluding other serious conditions. The cutaneous manifestation of AOSD varies, ranging from typical salmon-pink maculopapular rash to atypical rashes such as persistent purpuric papules and plaques, urticaria, dermographism-like rash, generalized erythema, and vesiculopustules on hands and feet [2–4].

## 2. Case Reports

### 2.1. Patient 1

**Figure 1:**
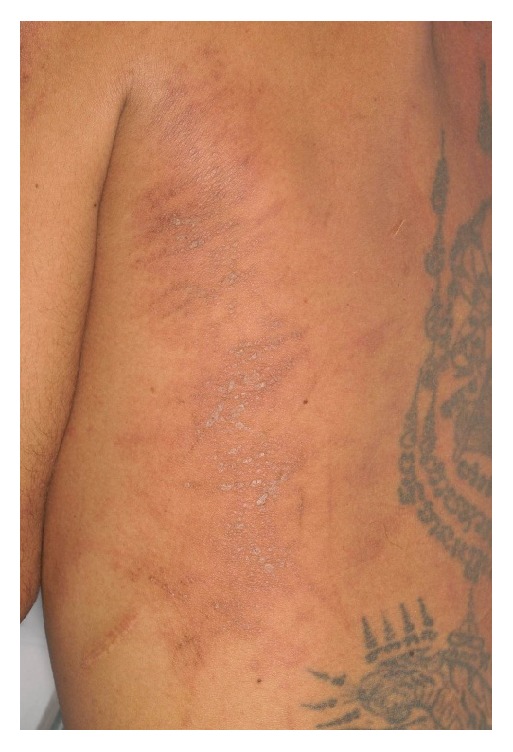
Linear, dermographism-like, and vague lichenoid papules.

A 36-year-old male with history of chronic symmetrical polyarthritis of hand joints was diagnosed with seronegative rheumatoid arthritis for five years. His current medicines were methotrexate 5 mg/week and chloroquine 250 mg/week. Prior to admission, the patient developed high-grade fever, rash with intense pruritus, interphalangeal joint pain, myalgia, and sore throat for three weeks. Antibiotics were given but no improvement was noted. On examination, his temperature was 39.5°C with mild pharyngeal hyperemia, hepatomegaly, and oligoarthralgia of the interphalangeal joints. Lung, spleen, and lymph node examinations were unremarkable. The persistent hyperpigmented plaques with excoriation and some scale were observed on his trunk, back, shoulders, and both thighs. He also had linear edematous erythematous wheal lesions, similar to dermographism in appearance on his back and shoulders (Figure 1). We observe no evanescent rash in this case. Laboratory findings are shown in Table 1. Chest radiography revealed bilateral perihilar interstitial infiltration. After excluding other conditions, he was diagnosed with AOSD. Prednisolone 60 mg/day was given with continued methotrexate 5 mg/week and increased dose of chloroquine from 250 mg/week to 250 mg/day. At the follow-up, corticosteroids were gradually decreased as symptoms, such as skin rash, fever, and arthralgia, showed improvement. Hepatitis, anemia, and interstitial infiltration of lung were also resolved.

### 2.2. Patient 2

**Figure 2:**
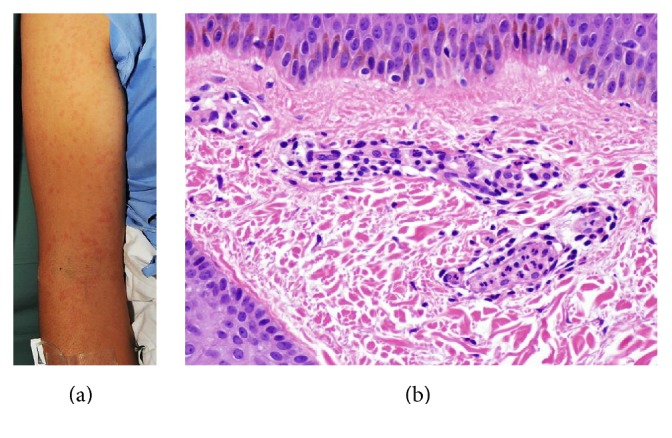
(a) Salmon-pink rash at forearm. (b) Normal epidermis with no necrotic keratinocytes. Superficial perivascular cells infiltration with neutrophils. No evidence of vasculitis (H&E, ×40).

A previously healthy 27-year-old woman developed high-grade fever, maculopapular rash with mild itching, weight loss, and polyarthralgia for 4 months. Hepatomegaly and bilateral symmetrical polyarthralgia of shoulders, wrists, knees, and phalangeal joints were also observed. The salmon-pink colored maculopapular rash was observed on her trunk, back, and extremities, with no residual hyperpigmentation observed when the rash subsided (Figure 2(a)). Skin biopsy was revealed as in Figure 2(b). Laboratory findings are shown in Table 1. Chest radiography was unremarkable. High-resolution computer tomography showed enlargement of multiple bilateral lymph nodes at axillary regions, with several subcentimeter lymph nodes noted at prevascular, periaortic, right paratracheal, and left upper paratracheal regions. Naproxen 500 mg/day, chloroquine 250 mg/day, and prednisolone 15 mg/day were prescribed. Fever, salmon-pink rash, and all other symptoms were subsided and completely resolved within a few months.

## 3. Discussion

Classic rash in AOSD is characterized by transient salmon-pink maculopapular rash, usually coexisting with daily high spiking fever [1]. However, atypical rashes have also been reported, such as persistent purpuric papules and plaques, urticaria, dermographism or linear pigmentation, generalized erythema, dermatomyositis-like plaques, vesiculopustules on hands and feet, prurigo pigmentosa-like plaques, and lichen amyloidosis-like hyperpigmented plaques [2–7]. Our first case presented with atypical dermographism-like and persistent hyperpigmented plaques. Recent studies have postulated that the pathophysiology of dermographism might be an underreported symptom of the Koebner phenomenon or related to mast cell degranulation [4, 5, 7].

AOSD has no pathognomonic histopathology. However, our review of the literature revealed highly distinctive findings of the biopsy specimens from persistent papules and plaques, including parakeratosis, scattered necrotic keratinocytes mostly in the upper half of the epidermis, and interstitial and perivascular neutrophilic infiltration in the papillary dermis with no evidence of vasculitis [6, 7]. On the other hand, the histopathology from urticarial evanescent rash shows normal epidermis, sparse perivascular, and interstitial neutrophilic infiltration with dermal edema [6, 7]. Patient 2 with maculopapular rash revealed the histopathology as superficial perivascular infiltration with neutrophils predominance, which can be found in, but not specific to, patient with AOSD.

Fever, arthralgia, typical rash, and leukocytosis are major criteria for Yamaguchi whereas sore throat, lymphadenopathy, hepatosplenomegaly, abnormal liver function tests, negative antinuclear antibodies, and negative rheumatoid factor are considered minor ones [1]. For Fautrel's criteria, spiking fever, arthralgia, transient erythema, pharyngitis, polymorphonuclear cells ≥ 80%, and glycosylated ferritin ≤ 20% are major criteria. Maculopapular rash and leukocytosis are minor criteria [1]. Our patients were diagnosed with AOSD according to both criteria, with other conditions such as infection, autoimmune diseases, and hematologic malignancy being excluded. Fever and joint pain are common symptoms; however, sore throat, myalgia, and weight loss can also be found. In addition, lymphadenopathy, splenomegaly, hepatitis, and/or hepatomegaly have frequently been reported. Rare interstitial lung infiltration can also develop, as observed in case patient 1 [1].

Some literatures suggested that autoimmunity mechanisms such as macrophage activation and inflammatory cytokines (e.g., IL-1, IL-6, IL-18, IFN-*δ*, and TNF-*α*), genetic factors, and infection may play a role of pathogenesis in AOSD [1, 2].

Recent studies have shown that most AOSD patients (93–100%) have negative rheumatoid factor (RF) and usually negative antinuclear antibodies (ANA) [1]. Given the presence of a positive ANA in patient 1, the patient was screened for systemic lupus erythematosus (SLE). Analysis revealed no SLE or specific autoantibodies for SLE in this case. In the second case, although the clinical features of AOSD were present, the patient also had positive RF. This suggests that despite the presence of positive RF and ANA, AOSD should not be dismissed.

Ferritin is usually higher in patients diagnosed with AOSD. If serum ferritin is 5 times above a normal level (normal range 30–400 ng/ml or above 1,000 ng/ml), it is suggested that the patient is having an AOSD, but with only 41–46% specificity [1]. Recent studies found that high serum ferritin level is usually related to disease activity and has been linked to chronic recurrent flares and reactive hemophagocytic lymphohistiocytosis [1, 8, 9]. Zeng et al. found that an elevated serum ferritin level, interstitial pneumonia, pleuritis, and unrecovered fever were still present after prescribing prednisolone 1 mg/kg/day for three days, foretelling poor prognosis [10]. Despite having high ferritin levels, both cases experienced good treatment outcomes. In our view, these positive outcomes might come from patients' good responses to prednisolone, which helped patients to recover from fever. In case 1 with extremely high ferritin level, there was a possibility of chronic recurrent flare in the future. Thus, close observation was needed.

The treatment for AOSD is based on the severity of the disease. Corticosteroids are useful in controlling active disease and inducing remission. NSAIDs are effective in curing mild cases and improving articular symptoms and fever. Steroid-sparing agents, such as methotrexate and hydroxychloroquine, were used and performed well in our cases. Biologic agents are an alternative treatment for use in the complicated and refractory cases [1, 11].

In conclusion, we report two cases with clinically compelling cutaneous lesions and clinical symptoms of AOSD. Dermographism-like lesions, which have rarely been reported, were found in patient 1. This case also showed a very high ferritin level and interstitial lung infiltration, indicating unfavorable prognosis. Patient 2 presented with typical rash but had positive rheumatoid factor. This suggests that although negative RF is one of the criteria used for the diagnosis of AOSD, a positive RF result does not exclude AOSD. Accordingly, other diagnostic criteria need to be considered and evaluated.

## Figures and Tables

**Table 1:** Laboratory findings from two patients presenting with adult-onset Still's disease.

Laboratory data	Patient 1	Patient 2
Hemoglobin (12–18 g/dL)	11.7	11.3
MCV/MCH (80–99 fL/27–37 pg)	79.8/26	84.8/27.8
WBC^*∗*^ count (4–11 × 10^3^/mm^3^)	29,020	25,040
Neutrophils (40–74%)	82.0	93.5
ESR^†^ (0–20 mm/hr)	86	94
CRP^‡^ (<5 mg/L)	N/A	89
Ferritin (30–400 ng/ml)	>100,000	13,753
AST^§^ (0–40 U/L)	385	100
ALT^||^ (0–41 U/L)	73	39
ALP^¶^ (40–130 U/L)	179	94
Albumin (3.5–5.2 g/dl)	2.9	3.9
LDH^*∗∗*^ (240–480 U/L)	6,883	872
Antinuclear antibodies	Homogeneous pattern titer 1 : 320; rim-like pattern; anti-cytoplasmic antibodies	Nucleolar pattern titer 1 : 100; fine-speckled pattern
Anti-double stranded DNA	Negative	Negative
Rheumatoid factor (<4.5 IU/ml = negative; 4.5–≤6.0 U/ml = borderline; >6.0 U/ml = positive)	Negative	23.36 (positive)
Anti-CCP^††^	Negative	N/A
Cultures	No growth (blood, urine)	No growth (blood)

^*∗*^White blood cell. ^†^Erythrocyte sedimentation rate. ^‡^C-reactive protein. ^§^Aspartate transaminase. ^||^Alanine transaminase. ^¶^Alkaline phosphatase. ^*∗∗*^Lactate dehydrogenase. ^††^Anti-cyclic citrullinated peptide antibody.
